# Dendritic cells pulsed with generated tumor cell lysate from *Phyllanthus amarus* Schum. & Thonn. induces anti-tumor immune response

**DOI:** 10.1186/s12906-018-2296-4

**Published:** 2018-08-06

**Authors:** Shimaa Ibrahim Abdelmenym Mohamed, Ibrahim Jantan, Mohd Azlan Nafiah, Mohamed Ali Seyed, Kok Meng Chan

**Affiliations:** 1 Drug and Herbal Research Centre, Faculty of Pharmacy, Universiti Kebangsaan Malaysia, Jalan Raja Muda Abdul Aziz, 50300 Kuala Lumpur, Malaysia; 2 Department of Chemistry, Faculty of Science and Mathematics, Universiti Pendidikan Sultan Idris, 35900 Tanjung Malim, Perak Malaysia; 3 Faculty of Medicine, University of Tabuk, Tabuk, 71491 Saudi Arabia; 4 Faculty of Health Sciences, Universiti Kebangsaan Malaysia, Jalan Raja Muda Abdul Aziz, 50300 Kuala Lumpur, Malaysia; 5 School of Pharmacy, Taylor’s University, Lakeside Campus, 47500 Subang Jaya, Selangor Malaysia

**Keywords:** *Phyllanthus amarus*, Dendritic cells, Immunotherapy, Tumor lysate, Anti-tumor, Immune response

## Abstract

**Background:**

Dendritic cells (DCs) are unique antigen presenting cells (APC) which play a pivotal role in immunotherapy and induction of an effective immune response against tumors. In the present study, 80% ethanol extract of *Phyllanthus amarus* was used to generate tumor lysate (TLY) derived from HCT 116 and MCF-7 cancer cell lines via induction of apoptosis. Monocyte-derived DCs were generated ex vivo from the adherent population of peripheral blood mononuclear cells (PBMCs). The generated TLY were used to impulse DCs to investigate its effect on their cellular immune functions including antigen presentation capacity, phagocytic activity, chemotaxis capacity, T-cell proliferation and cytokines release.

**Methods:**

The effect of *P. amarus*-generated TLY on DCs maturation was evaluated by determination of MHC class I, II and CD 11c expression as well as the co-stimulatory molecules CD 83 and 86 by using flow cytometry. The phagocytic capacity of TLY-pulsed DCs was investigated through FITC-dextran uptake by using flow cytometry. The effect on the cytokines release including IL-12, IL-6 and IL-10 was elucidated by using ELISA. The migration capacity and T cell proliferation activity of pulsed DCs were measured. The relative gene expression levels of cytokines were determined by using qRT-PCR. The major constituents of *P. amarus* extract were qualitatively and quantitatively analyzed by using validated reversed-phase high performance liquid chromatography (HPLC) methods.

**Results:**

*P. amarus*-generated TLY significantly up-regulated the expression levels of MHC class I, CD 11 c, CD 83 and 86 in pulsed DCs. The release of interleukin IL-12 and IL-6 was enhanced by TLY-DCs at a ratio of 1 DC: 3 tumor apoptotic bodies (APO), however, the release of IL-10 was suppressed. The migration ability as well as allogeneic T-cell proliferation activities of loaded DCs were significantly enhanced, but their phagocytic capacity was highly attenuated. The gene expression profiles for IL-12 and IL-6 of DCs showed increase in their mRNA gene expression in TLY pulsed DCs versus unloaded and LPS-treated only DCs.

**Conclusion:**

The effect of *P. amarus*-generated TLY on the immune effector mechanisms of DCs verified its potential to induce an in vitro anti-tumor immune response against the recognized tumor antigen.

## Background

There are several treatment modalities of cancer including surgery, radiation therapy, chemotherapy, hormonal therapy, and immunotherapy. Treatment modalities can be used alone or in combination based on the stage of the disease and also the general health condition of the patient. Most of cancer treatment modalities were proved to be ineligible in spite of their moderate progression. This was assumed due to the inadequate immune response against the progressive tumors mainly in the development of effector T cell responses. Cancer immunotherapy represents a novel approach that destroys the existing tumor cells as well as develops a long-lasting immunity which will prevent tumor relapse [1]. It aims to induce the immune system to target the tumor antigens and proteins that are specifically expressed by the tumor cells. Tumor associated antigens (TAA) are normal proteins but overexpressed on the tumor cells. Dendritic cells (DCs) are the most potent antigens presenting cells (APC) and act as a link between innate and adaptive immune systems [2]. Immature DCs possess the capacity to capture and uptake antigens which results in the maturation of dendritic cells and then followed by their migration to the lymphoid organs [3]. Mature DCs process and present the TAA to naïve T cells through major histocompatibility complex (MHC) class I and II that leads to activation and clonal expansion of T lymphocytes mainly into CD 4^+^ helper T cell and CD8^+^ cytotoxic T cells. Furthermore, mature DCs are characterized by the high expression level of MHC as well as co-stimulatory molecules such as CD 80, CD 83 and CD 86 that stabilize the interaction between DCs and naïve T lymphocytes for induction of antitumor immune response [4]. This is in addition to the release of pro-inflammatory cytokines such as interferon (IFN)-γ and interleukin (IL)-12 that activate Th1 response, which catalyzes the activation of cytotoxic T lymphocyte (CTL) [5]. Activated CTL can effectively migrate and infiltrate the tumor microenvironment to directly attack the tumor cells by induction of apoptosis as well as via release of perforin and granzymes [6]. Additionally, DCs possess the ability to induce NK cells and B cells. This dual role in both the innate and adaptive immune systems derived into the possibility of using DCs in immunotherapy in combination with alternative treatment modalities. The using of tumor lysate pulsed-DCs represents a new approach to boost the patient immunity and induces an effective cytotoxic immune response against the tumor cells, which can easily escape the immune surveillance [7]. The apoptotic cells were proved to be an efficient source of antigens that can be recognized by the immune system [8]. Moreover, the protein fragments derived from phagocytosed cells are well presented more than the processed by MHC II products of DCs [9].

In addition to the induction of antitumor immune response, the cancer immunotherapy functions through the alteration of tumor microenvironment by the release of pro-inflammatory cytokines such as IL-12, IL-6, and TNF-α. IL-12 is produced mainly by DCs and stimulates the cytotoxic activity of natural killer cells (NKs) as well as the clonal expansion of naïve T lymphocytes into CD4^+^ helper and CD8^+^ cytotoxic T cells (CTL) [10, 11]. Furthermore, the release of tumor necrosis factor (TNF)-α, IL-1α/β and IL-6, can induce an immune response against the tumor and increase the production of reactive oxygen species (ROS) and reactive nitrogen species (RNS) by the immune cells infiltrating the tumor microenvironment that have a destructive effect on the tumor cells [12, 13]. Several clinical studies have been conducted to evaluate the effectiveness of DCs based vaccines to induce tumor-antigen specific immune response against various types of cancer including prostate cancer [14], ovarian cancer [15], renal cell cancer [16], metastatic melanoma cell cancer [17], pancreatic cancer [18] and glioblastoma [19]. Moreover, there is a tendency in the recent trials to use tumor antigen obtained from patient’s tumor sample.

*Phyllanthus amarus* has been widely used as a traditional and alternative medication in the treatment of various diseases including diarrhea, jaundice, kidney disorders, influenza, diabetes, liver ailment, fever, scabies, ulcers and wound [20–23]. The hairy root methanol extract of *P. amarus* was found to exhibit anti-proliferative effect on MCF-7 human breast cancer cells via induction of apoptosis by increasing ROS level as well as the reduction in mitochondrial membrane potential [24]. *P. amarus* induced strong cytotoxic and apoptotic activities on MCF-7 and human lung cancer A549 cells alongside with anti-metastasis effect [25]. Therefore, in the previous studies, the anticancer effect of *P. amarus* has been reported through anti-proliferative, apoptotic, anti-metastatic and anti-angiogenesis activities. However, there are no reports available on the effect of pulsing of DCs against tumor antigen generated by *Phyllanthus* spp. Thus, the rationale behind our proposed study was that using of *P. amarus* to elicit apoptosis in the tumor cells and create a collection of TAAs in the form of dead and dying cellular debris that could activate APC mainly DCs. Additionally, the cellular immune functions (i.e. antigen presentation capacity, phagocytic activity, chemotaxis, T-cell proliferation and cytokines release) were investigated by using *P. amarus*-generated tumor lysate on DCs grown ex vivo*.* This finding can contribute to the development of a novel DCs based vaccine strategy by using natural immunomodulators for colon and breast cancer.

## Methods

All experiments by using human whole blood were carried out under a protocol approved by the Human Ethical Committee of Universiti Kebangsaan Malaysia (Approval no: UKM PPI/111/8/JEP-2017-335).

### Collection of plant material

The whole plant of *P. amarus* was obtained from Marang, Kuala Terengganu, Malaysia in the month of June 2012. The plant was authenticated by Dr. Abdul Latif Mohamad of the Faculty of Science and Technology, Universiti Kebangsaan Malaysia (UKM), and a voucher specimen (UKMB 30078) was deposited at the Herbarium of UKM, Bangi, Malaysia. The collection of plant samples did not involve endangered or protected species, and the study was carried out at the Drug and Herbal Research Centre, Faculty of Pharmacy, UKM. The whole plant of *P. amarus* (1 kg) was ground and extracted with 80% EtOH (3 × 3 L) at room temperature for 72 h, then filtered through Whatman® Grade1 filter paper (Sigma-Aldrich Corp). The filtrate was collected, and excess solvent was evaporated under reduced pressure using a rotary evaporator at temperature between 55 and 60 °C. The yield of extract obtained was 108 g (10.8% *w*/w). The extract was examined for the endotoxin contamination by using E-Toxate assay kit (Sigma-Aldrich Co. LLC) according to manufacturer’s protocol.

### High performance liquid chromatography analysis of 80% ethanol extract of *Phyllanthus amarus*

High performance liquid chromatography (HPLC) analysis and validation were performed based on the chromatographic conditions described by Jantan et al. [26]. Briefly, the HPLC analysis was performed using the following conditions: reverse-phase C-18 column (250 mm × 4.6 mm ID, 5 m, XBridge™; Waters Corporation, Milford, MA, USA), and photodiode array (PDA) detector (Waters 2998) of wavelength ranging from 205 to 270 nm. Identification and quantification of components of the extracts and standard compounds including gallic acid, ellagic acid, corilagin, geraniin, niranthin, phyltetralin, isolintetralin, phyllanthin, and hypophyllanthin were performed using two different chromatographic conditions; method 1 and method 2, as described in our previous study [26]. HPLC analyses of the extracts and standard solutions of compounds (gallic acid, ellagic acid, corilagin, geraniin, niranthin, phyltetralin and isolintetralin) were performed using method 1. The identification of each compound was carried out by comparing the retention times and ultraviolet-visible (UV-Vis) spectra of the peaks with those of the standard compounds. Identification and quantification of the phyllanthin and hypophyllanthin were carried out based on the chromatographic conditions described in method 2. The validation of HPLC for the standardization of the extract was carried out by determination of linearity, precision, and limits of quantification (LOQ) and detection (LOD).

### Cell lines and culture

Cancerous cells MCF-7 human breast cancer and HCT 116 human colon cancer cell lines were obtained from American Type Culture Collection (USA). The cells were cultured and maintained in Dulbecco’s modified Eagle’s medium (DMEM) (Gibco Co. USA) supplemented by 10% fetal bovine serum (FBS) (Sigma, St. Louis, MO, USA) and 1% antibiotic (streptomycin 200 μg/mL and penicillin 100 units/mL) (Gibco Co. USA). Cells were incubated at 37 °C in 5% CO_2_ environment and then detached and harvested at confluence 80–90% by trypsinization with trypsin-EDTA (Sigma, St. Louis, MO, USA).

### Preparation of *Phyllanthus amarus* generated tumor lysate

Apoptotic bodies were prepared and purified. Briefly, the cells (passage # 5) were treated with 80% ethanol extract of *P. amarus* at a concentration of 1000 μg/mL. Floating dead cells were collected everyday by centrifugation at 125 xg for 10 min and stored at 4 °C until they were subjected for 5 cycles of freeze-thaw. Purified apoptotic bodies were stained with FITC- Annexine V apoptosis detection kit (BD Pharmingen, BD Bioscience, USA) for determination of apoptosis by FACScan analysis. The apoptotic cells were suspended in 2 mL of HBSS and lysed by 5 freezes (liquid nitrogen)–thaw (at room temperature) cycles. Total cell disruption was microscopically validated using trypan blue staining. After sonication for 10 min, lysate was centrifuged at 15000 xg for 15 min at 4 °C and stored in aliquots at − 80 °C until use.

### Generation of monocyte-derived dendritic cells ex vivo

All experiments by using human blood were carried out under a protocol approved by the Universiti Kebangsaan Malaysia Research Ethics Committee (No. UKM PPI/111/8/JEP-2017-335). DCs were generated from freshly isolated peripheral blood monocytes (PBMCs). In brief, PBMCs were isolated from peripheral blood of healthy donors by Lymphoprep® separation medium (Axis- Shield Pc-AS, Oslo, Norway). Cells were allowed to adhere by incubation for 1 h at 5% CO_2_ and 37 °C in an appropriate amount of PromoCell monocyte attachment medium (Promo-Cell GmbH, Heidelberg, Germany) at a density of 2–3 million/cm^2^. The adherent cells were washed three times with warm monocyte attachment medium by swirling the vessel and aspirating the supernatant. The cells were cultured in an appropriate amount of PromoCell DCs generation medium DXF supplemented with GM-CSF (1000 units/mL) and IL-4 (1000 units/mL) (Promo-Cell GmbH, Heidelberg, Germany) then incubated at 37 °C and 5% CO_2_. The generation of immature dendritic cells was characterized by CD14^−^, HLA-DR bright, CD83^+^, and CD 86^+^ phenotypical expression using flow cytometry analyses. On day 6, the cells were seeded in 6-well plates at a density of 1 × 10^6^ cells/mL and co-cultured with lysate generated from *P. amarus*-treated tumor cells at different ratios (1:1, 1:3, 1:5 and 1:7) DC: APO for 4 h. DCs were subsequently activated with LPS from *Escherichia coli* strain (Sigma, St. Louis, MO, USA) at 1 μg/mL for 48 h which results in the generation of mature tumor lysate pulsed dendritic cells (TLY-DCs). However, LPS-only stimulated DCs were generated by activation of immature DCs with LPS only at a concentration of 1 μg/mL for 48 h. The cell viability was evaluated by using by trypan blue exclusion method.

### Determination of the phagocytosis of *Phyllanthus amarus*-treated damaged tumor cells by immature dendritic cells

For induction of an effective immune response, DCs need first to uptake tumor-derived material before processing and presenting the antigen to T lymphocytes. Therefore, we investigated whether monocytes-derived-DCs (mo-DCs) were able to endocytose *P.amarus*-treated damaged tumor cells remnants and fragments. To validate this, both HCT 116 (colon cancer) and MCF-7 (breast cancer) cells were stained with a fluorescent dye CFSE [5, 6-carboxyfluorescein diacetate succinimidyl ester] (BD Pharmingen, BD Bioscience, USA) that gives a strong and stable green fluorescence. CFSE-labeled tumor cells were seeded in 6 well plates and incubated with 80% ethanol extract of *Phyllanthus amarus* at a concentration of 1000 μg/mL. The tumor cells fragments were prepared by 5 freeze and thaw cycles. The CFSE-labeled tumor cells remnants were co-cultured with DCs at ratio 1: 3 (DCs: APO) in culture media and incubated for 24 h at 37 °C and 5% CO_2_. The non-ingested debris was removed and the uptake of fluorescent labelled tumor cells remnants by DCs (stained with APC-H7 conjugated antihuman monoclonal antibody against HLA-DR) was determined by flow cytometer analysis.

### Endocytic activity of tumor lysate pulsed dendritic cells

Endocytic activity of DCs was assessed by the uptake of fluorescein iso-thiocyanate FITC-dextran (Sigma, St. Louis, MO, USA). Immature, mature and LPS-only stimulated dendritic cells were cultured in the complete culture medium containing FITC-dextran at a concentration of 0.5 mg/mL and incubated for 30 min at 5% CO_2_ and 37 °C (negative control was incubated on ice). After the incubation time, cells were extensively washed for 3 times using phosphate buffered saline (PBS) (Sigma, St. Louis, MO, USA) and the uptake of FITC-dextran was analyzed by flow cytometry analysis.

### Phenotypic characterization

In order to determine the maturation and antigen presentation capacity of mo-DCs after engulfment of *P. amarus*- generated tumor lysate, 1 × 10^6^ /mL cells were stained with anti-human monoclonal antibodies PE-Cy7- conjugated anti-CD11c, Per-CP conjugated anti-HLA-I, APC-conjugated anti-CD 86 and APC-H7- conjugated anti-HLA-DR and anti-CD 14 for 15 min at room temperature in PBS containing 2% FBS. After the incubation time, the cells were washed with 1 mL cold PBS and analyzed by flow cytometer BD FACS Canto II. All anti-human monoclonal antibodies against HLA-DR, HLA-I, CD 86, CD 83, CD 11c and CD 14 were acquired from BD Pharmingen (BD Bioscience, USA).

### Cytokine release

For the measurements of IL-12 P40, IL-6, and IL-10, mo-DCs were pulsed first with PA generated tumor lysate. DCs were treated with tumor lysate at ratios 1:1 and 1:3 of DCs: APO for 4 h and then stimulated with LPS (1 μg /mL) for 48 h at 37 °C in 5% CO_2_. The cytokines in the supernatants were determined using enzyme-linked immunosorbent (ELISA) kits (R&D System, Minneapolis, MN, USA) according to the manufacturer’s instructions. The concentrations were calculated from the standard curves.

### Migration assay

Chemotaxis activity of tumor lysate pulsed DCs (1:1 and 1:3) was assessed by using a commercially available 96-well cell migration assay kit (Cytoselect 96-Well Cell Migration Assay, Cell Biolabs, INC.) of 5 μm pore size. The cell suspension comprising of 1 × 10^6^ cells/mL was prepared in serum-free media. One hundred and fifty μL of media containing CCL 21 (at a concentration of 250 ng/mL) was added to the membrane chamber. CCL21 (R&D System, Minneapolis, MN, USA) is a lymph node (LN) secreted chemokine and acts as chemoattractant agent for DCs. After covering the plate, it was placed in 37 °C incubator for 3 h. Prior to the end of the incubation time, 150 μL of cell detachment solution was transferred into the wells of a clean 96-well cell harvesting tray. The 96-well cell migration plate was then carefully removed from the incubator, followed by the separation of the membrane chamber from the feeder tray. The media from the top of the membrane chamber were removed by aspiration. The membrane chamber was then placed into the cell harvesting tray containing 150 μL of cell detachment solution, followed by incubation for 30 min at 37 °C. The cells from the underside of the membrane were dislodged by tilting the membrane chamber in the cell detachment solution. Fifty μL of 4X Lysis buffer/CyQuant GR dye solution was added to each well, followed by incubation at room temperature for 20 min. At the end, 150 μL of the mixture was transferred to the 96-well plate for measurement of fluorescence in the fluorescence plate reader at 480 nm/ 520 nm.

### T cell proliferation assay

Allogeneic T cells enriched fractions were obtained as 1 h non-adherent cells of PBMCs prepared from the buffy coats. The capacity of mature DCs to induce the proliferation of allogeneic T-cells was assessed. DCs (2 × 10^5^ cells/mL) were harvested and co-cultured with allogeneic non-adherent mononuclear in 96 well round bottom plates at different ratios 1:20, 1:40, 1:80 and 1:160 (DCs: T cells) in triplicates. Phytohaemagglutinin (PHA) (20 μg/mL) (Sigma, St. Louis, MO, USA) was used as positive control. After incubation at 37 °C and 5% CO_2_ for 4 days, the cells were pulsed with 1 μCi/well of [H]^3^ Thymidine and incubated for further 18 h at 37 °C and 5% CO_2_. After incubation, the cells were harvested by using glass fiber filters. The harvested cells were dissolved in 2.5 mL of liquid scintillation cocktail and the thymidine incorporation was determined by using liquid scintillation counter.

### Quantitative PCR

The effect *P. amarus* generated tumor lysate on the gene expression of IL-12, IL-6, IL-10 in dendritic cells was examined by real-time polymerase chain reaction (RT-PCR). Monocyte-derived dendritic cells were seeded in 24-well plates at a density of 1 × 10^6^ cells/mL. Dendritic cells were pretreated with *P. amarus* generated tumor lysate at ratio 1DCs: 3 tumor cells for 4 h before stimulation with LPS at 1 mg/mL for 48 h. The total RNA from iDCs, TLY-DCs and LPS treated DCs was isolated and purified using RNeasy Micro Kit according to the manufacturer’s protocol. The quantity and integrity of the extracted RNA were analyzed using Nano-Drop spectrophotometer (Thermo Scientific, Switzerland). Total RNA was reverse transcribed to cDNA by using Quantinova ™ Reverse Transcription kit according to the manufacture’s protocol (Qiagen, UK). The cDNA was amplified by using the following primers IL-12 P40 (For- CGGTCATCTGCCGCAAA Rev-TGCCCATTCGCTCCAAGA), IL-10 (For-GGTGATGCCCCAAGCTGA Rev-TCCCCCAGGGAGTTCACA), IL-6 (For-AGCCACTCACCTCTTCAGAACGAA Rev-CAGTGCCTCTTTGCTGCTTTCACA), and GAPDH (For-AGCCTCAAGATCATCAGCAATG Rev-CACGATACCAAAGTTGTCATGGA). Quantitative PCR was performed on cDNA by using SYPER GREEN MASTER MIX (Quanti-Nova ™ SYPER Green PCR Kit, Qiagen, UK) on Bio-Rad CFX- Real-Time PCR System Thermal Cyclers. A total of 40 cycles was performed. The relative fold change between samples was determined by using the comparative cycle threshold method (2^−ΔΔCt^). The gene expression was normalized against the housekeeping gene GAPDH mRNA expression.

### Statistical analysis

All the experiments were performed on three independent healthy donors and data presented as the mean ± standard error of mean (SEM). All the data were statistically analyzed using Graph Pad Prism 5 software (Graphpad Software, Inc., La Jolla, CA, USA) by one-way analysis of variance (ANOVA) to determine the mean difference between groups, followed by Dunnett’s test *P* ≤ 0.05 was considered statistically significant.

## Results

### HPLC qualitative and quantitative analyses of 80% ethanol extract of *Phyllanthus amarus*

**Fig. 1:**
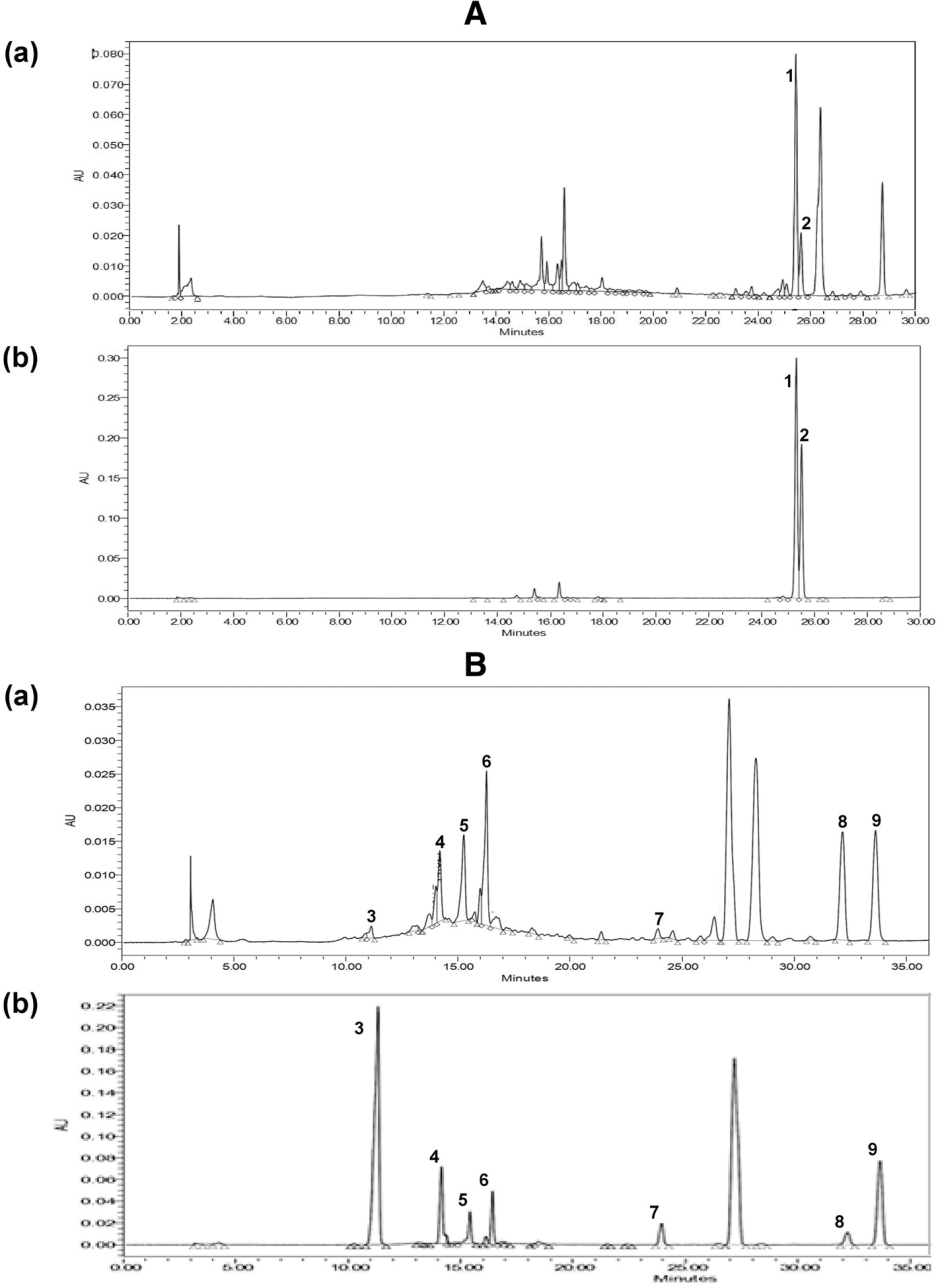
HPLC analyses of 80% ethanol extract of *Phyllanthus amarus*. **a** Representative HPLC chromatograms of (a) 80% ethanol extract of *Phyllanthus amarus* (b) mixture of standards for identification and quantification of (1) phyllanthin (RT 25.354 min) and (2) hypophyllanthin (RT 25.547 min). **b** Representative HPLC chromatograms of (a) 80% ethanol extract of *Phyllanthus amarus* (b) mixture of standards for identification and quantification of (3) gallic acid at (RT 11.155 min), (4) geraniin at (RT 14.204 min), (5) corilagin at (RT 15.273 min), (6) ellagic acid at (RT 16.283 min), (7) niranthin at (RT 23.933), (8) phyltetralin at (RT 32.157), and (9) isolintetralin at (RT 33.628)

The chromatograms of reversed-phase HPLC of the 80% ethanol extract of *P. amarus* showed nine major compounds (gallic acid, ellagic acid, geraniin, corilagin, niranthin, phyltetralin, isolintetralin, phyllanthin, and hypophyllanthin) (Fig. 1a and b). Quantitative determination of the major compounds by HPLC indicated that ellagic acid was the most abundant at concentration of 218.833 μg/mL followed by phyllanthin (170.69 μg/mL), and corilagin (138.689 μg/mL) (Table 1). The calibration curves were plotted for the standard solutions of different compounds showed correlation coefficients (r^2^) of ≥0.996. The reproducibility of the results was confirmed by the relative standard deviations (% RSDs) of the mean area under the peak and mean retention time by inter day and intra-assay precision assays.

**Table 1:** Quantification of the major compounds found in *Phyllanthus amarus* (μg/mL) by using HPLC quantification assay

No.	Compound	Concentration (μg/mL)	Retention time (min)
Method 2			
1	Phyllanthin	170.69	25.354
2	Hypophyllanthin	76.863	25.547
Method 1			
3	Gallic acid	53.732	11.155
4	Geraniin	96.569	14.204
5	Corilagin	138.68	15.273
6	Ellagic acid	218.833	16.283
7	Niranthin	46.428	23.826
8	Phyltetralin	122.46	32.157
9	Isolintetralin	119.018	33.628

### Generation and pulsing of immature DCs with tumor lysate

**Fig. 2:**
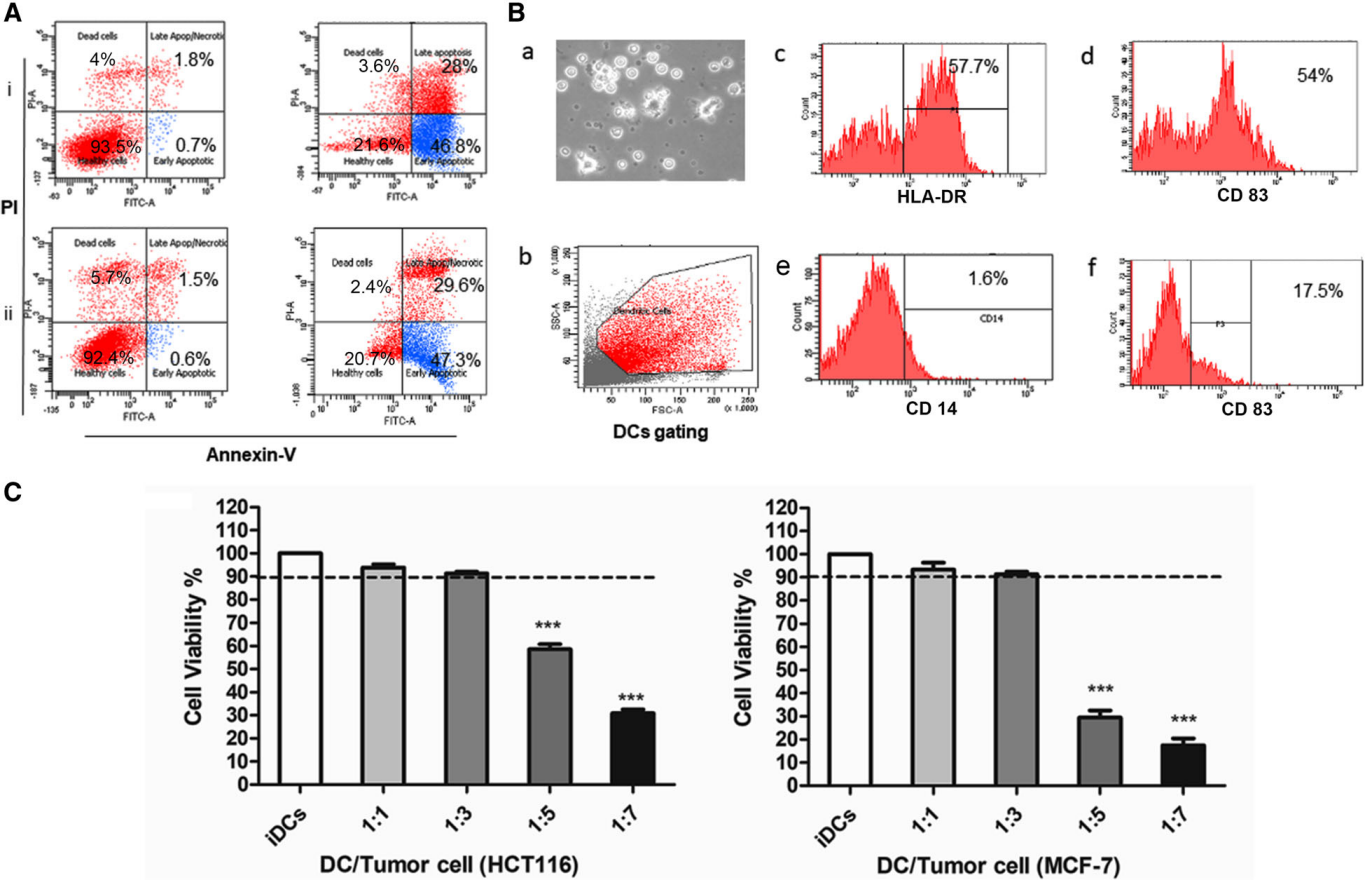
Generation of tumor lysate and monocyte-derived dendritic cells. **a** Flow cytometric analysis of the apoptotic effect of 80% ethanol extract of *Phyllanthus amarus* on (i) HCT-116 and (ii) MCF-7. **b** Characterization of the generated immature monocyte-derived DCs ex vivo at day 6 (a) microscopically, (b) representative gating strategy, and (c-f) flow cytometric analysis of HLA-DR^+^, CD 83^+^, CD 14^−^ and CD 86^+^ expression by iDCs. **c** The cell viability assay of DCs treated with PA generated tumor lysate (HCT 116 and MCF-7 TLY) by trypan blue exclusion method after 48 h. The data are representative of three experiments comprising three different healthy donors and analyzed by one-way ANOVA followed by Dunnett’s test. ***P < 0.001. Mean ± SEM. are shown. DCs, Dendritic cells; PA, *Phyllanthus amarus*; Ann V, Annexin V; PI, Propidium iodide

The induction of apoptosis after treatment with *P. amarus* in HCT 116 and MCF-7 at 1000 μg/mL for 24 h was determined by Annexin V-FITC/PI dual staining assay (Fig. 2a). Generation of monocyte-derived dendritic cells was highly reproducible. The generated dendritic cells were characterized microscopically as a cluster of cells with veil like-processes. Additionally, the immature dendritic cells expressed CD 14 ^-^, HLA-DR^+^ (57.7%), CD 86 ^+^ (54%), and CD 83 ^+^ (17.5%) that were determined by flow cytometry analyses (Fig. 2b). At day 6, immature dendritic cells were co-cultured with tumor lysate that was generated by five freeze and thaw cycles from *P. amarus*-treated HCT 116 human colon cells and MCF-7 adenocarcinoma breast cancer cells. The cell viability of tumor lysate dendritic cells was determined by trypan blue exclusion method and the cells were counted by hemocytometer. The treatment of DCs with tumor apoptotic bodies (APO) at ratios 1:1 and 1:3 (DCs: APO) was found to be non-toxic (cell viability ≥90%), however, at ratio 1:5 or more influenced the cell viability (Fig. 2c).

### Phagocytic capacity of the generated DCs to uptake the tumor lysate

**Fig. 3:**
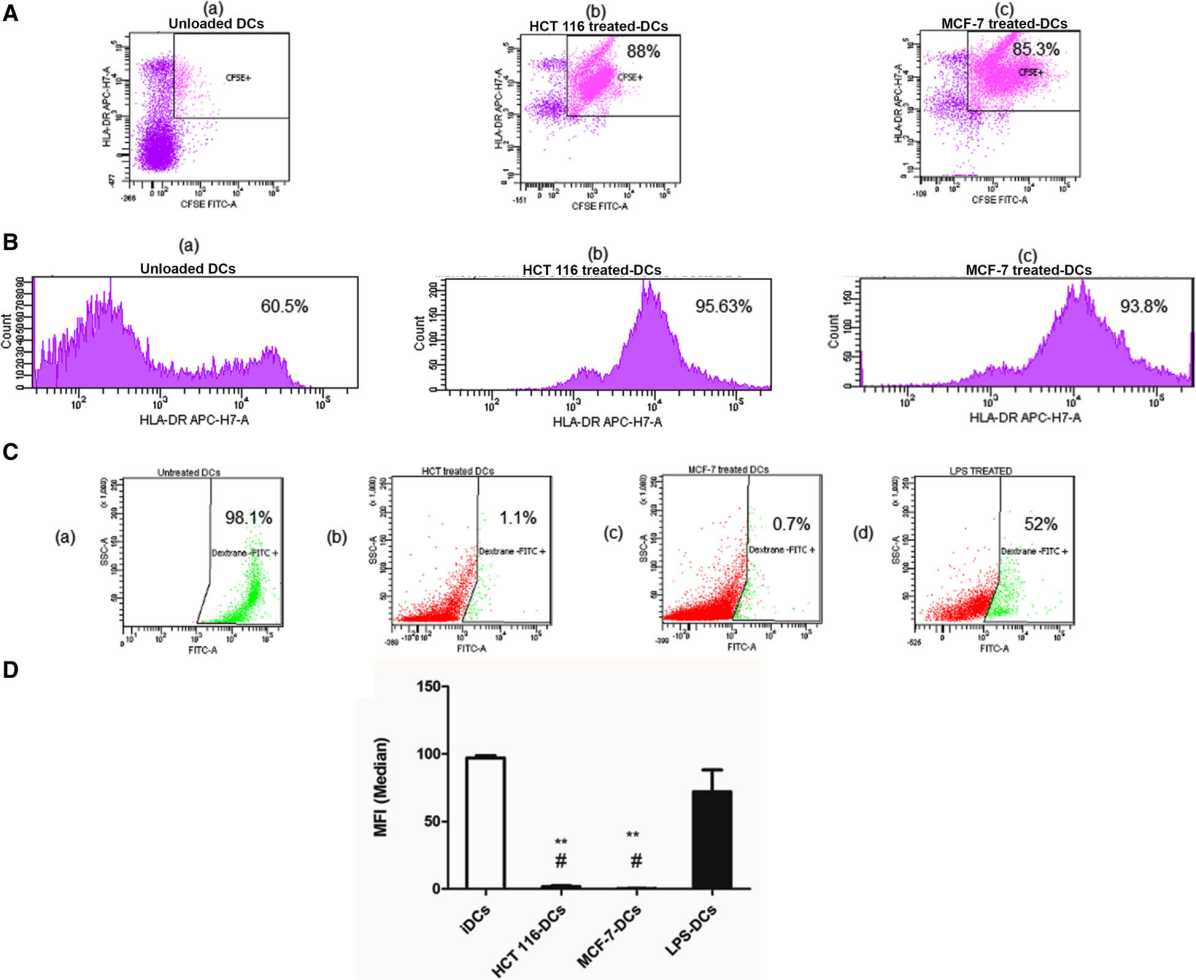
The phagocytic capacity of dendritic cells towards CFSE-labeled tumor debris and FITC-dextran. **a** Flow cytometric analysis indicates the percentage of DCs population with positive uptake of CFSE stained tumor cell remnants in (a) iDCs, (b) HCT 116 treated-DCs and (c) MCF-7 treated-DCs. **b** Identification of DCs as high HLA-DR^+^ population for (a) unloaded iDCs, (b) HCT 116-treated-DCs and (c) MCF-7-treated-DCs. **c** Representative FACs analysis data for FITC-dextran uptake by (a) iDCs, (b) HCT 116-TLY-pulsed-DCs, (c) MCF-7-TLY pulsed-DCs, and (d) LPS–stimulated only DCs. **d** The figure represents the statistically transformed data. ** Indicates significant difference (** *P* < 0.01) for TLY-treated-DCs versus iDCs. ## Indicated significant difference (## *P* < 0.01) of TLY-DCs versus LPS-stimulated DCs. The data are representative of three experiments comprising three different healthy donors and analyzed by one-way ANOVA followed by Dunnett’s test. iDCs, immature dendritic cell; TLY, tumor lysate; CFSE, Carboxy-fluorescein succinimidyl ester

The phagocytic capacity of the generated DCs towards HCT 116 human colon and MCF-7 human breast cancer cell debris was studied. The uptake of the carboxyfluorescein succinimidyl ester (CFSE)-labelled tumor cell remnants was monitored by flow cytometry as a proportion of HLA-DR ^+^ DCs acquired green fluorescence. The data showed that DCs have a high capacity to endocytose the CFSE-labeled tumor debris with 88 and 85.3% for HCT 116 and MCF-7, respectively (Fig. 3a). The percentage of phagocytosis represented the proportion of viable DCs (HLA-DR ^+^) which obtained the green fluorescence (CFSE^+^) (Fig. 3b).

### Decrease in the endocytic capacity of tumor lysate pulsed DCs

Generally, DCs express mannose receptor on their surface that allows the dextran phagocytosis. This active uptake can reflect the antigen endocytic capacity of DCs. The data demonstrated that unloaded immature DCs exhibited the highest capacity to incorporate FITC-dextran up to 98.1 ± 1.6%. However, in mature tumor lysate-pulsed DCs, the cells lost their endocytic capacity to incorporate dextran followed the maturation and uptake of tumor lysates (1.1 ± 0.65 for HCT 116 TLY-DCs and 0.7 ± 0.15% for MCF-7 TLY-DCs) as shown in Fig. 3c and d. LPS-stimulated dendritic cells showed moderate dextran uptake (52 ± 3.52%). The endocytic capacity of DCs was negatively correlated with the cell activation and uptake of CFSE-labeled tumor lysate.

### Upregulation in the DCs surface marker expression

**Fig. 4:**
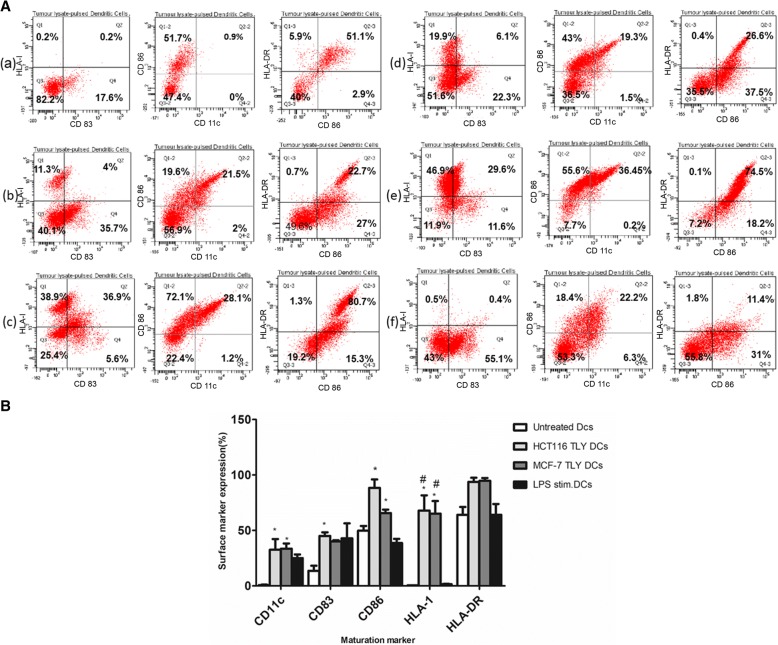
Upregulation in the phenotypic expression of maturation markers in tumor lysate-pulsed dendritic cells. **a** Flow cytometric analysis of phenotypic HLA-DR, HLA-I, CD 83, 86 and CD 11c expression in (a) unloaded immature DCs, HCT-116-TLY-DCs at (b) 1:1, (c) 1:3 ratios, MCF-7-TLY at (d) 1:1, (e) 1:3 ratios, and (f) LPS-stimulated-only DCs. **b** The statistically transformed data show the significant increase in the expression levels of the maturation markers HLA-I, CD 83, 86 and CD 11c in tumor lysate pulsed dendritic cells over immature non-pulsed DCs (* *P* < 0.05) and LPS-only stimulated DCs (#*P* < 0.05). The data are representative of three experiments comprising three different healthy donors and analyzed by one-way ANOVA followed by Dunnett’s test. iDCs, immature dendritic cells; TLY, tumor lysate; LPS, Lipopolysaccharide

In the phenotypic characterization of tumor lysate pulsed DCs (TLY-DCs), the data showed that the surface expression of MHC molecules, HLA-DR and HLA-I, were markedly increased in comparison with the immature unloaded DCs and LPS-stimulated DCs at ratio 1:3 (DC: APO). Whereas, the mean HLA-DR surface expression rate upregulated from 64.07 ± 4.02 up to 93.8 ± 3.7 and 94.7 ± 2.55% in HCT 116-TLY–DCs and MCF-7-TLY-DCs, respectively (Fig. 4a). The results revealed that the immature DCs expressed low levels of HLA-I and CD 11c, however, in pulsed dendritic cells the surface expression levels of HLA-1 and adhesion molecule CD11c were significantly elevated. Moreover, the expression levels of co-stimulatory molecules, CD 83 and CD 86 that stabilize the interaction between dendritic cells and T lymphocytes, were dramatically increased in comparison with the immature DCs as well as LPS-only stimulated DCs. In HCT 116 TLY–DCs, the maturation and expression of co-stimulatory CD 86 (88.35 ± 6.45%) and CD 83 (45.05 ± 3.25%) molecules were more efficient than MCF-7 TLY-DCs with the mean expression values of CD 86 and CD 83 of 65.55 ± 3.25% and 40.15 ± 1.05%, respectively (Fig. 4b).

### IL-12, IL-6, and IL-10 productions by dendritic cells

**Fig. 5:**
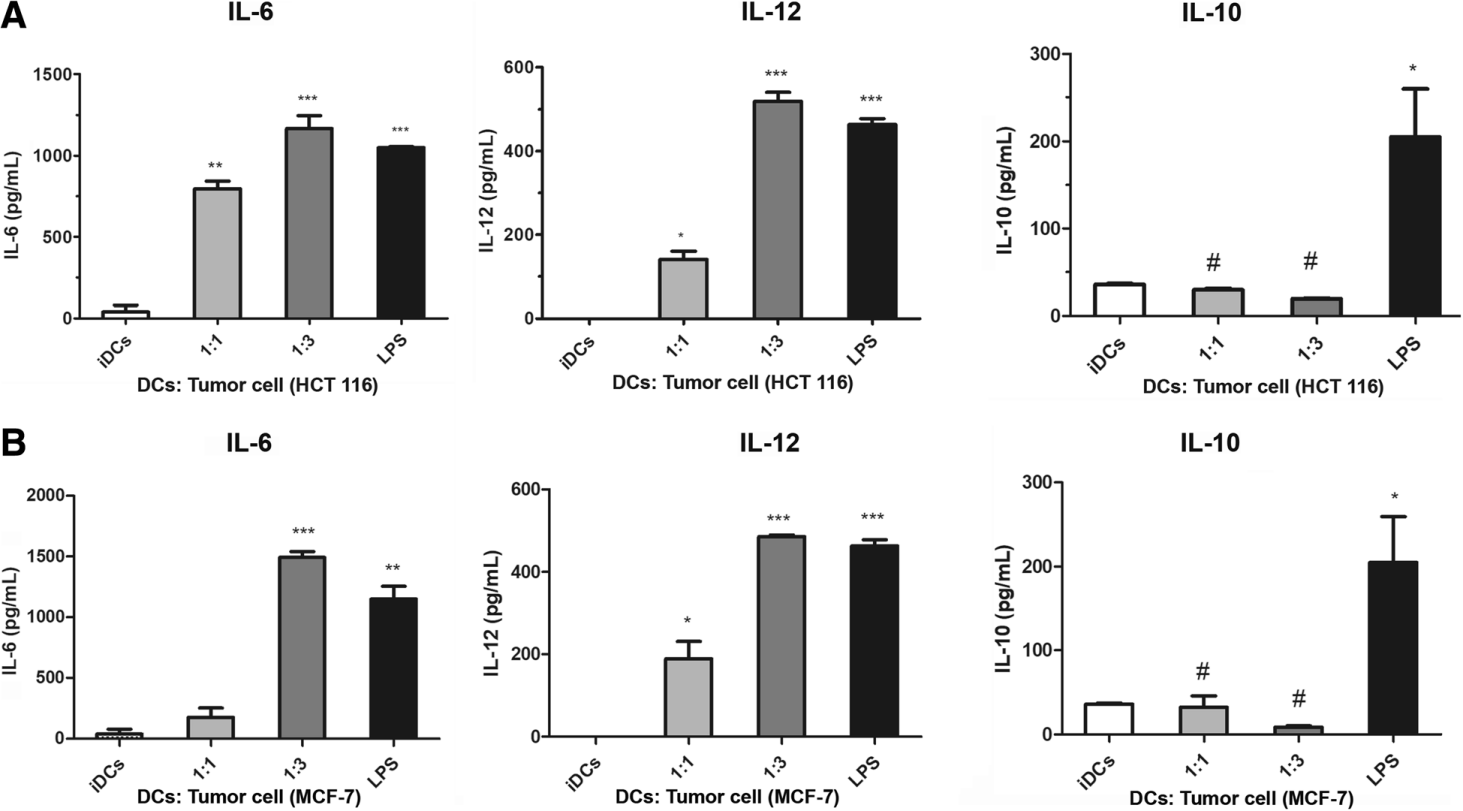
Increase of IL-12 P40, IL-6, and decrease of IL-10 cytokines release by tumor lysate-loaded dendritic cells. ELISA was used to quantify the cytokines concentrations in the supernatants of unloaded DCs, **a** HCT116-TLY-pulsed DCs, **b** MCF-7-TLY-pulsed DCs and LPS-only-stimulated DCs. Each point represents the mean ± SEM of three independent experiments comprising three different healthy donors. The data were statistically analyzed by one-way ANOVA followed by Dunnett’s test *P < 0.05, ** *P* < 0.01, *** *P* < 0.001. DCs, dendritic cells; TLY, tumor lysate; ELISA; enzyme-linked immunosorbent assay

High level of IL-12 P 40 was detected in the supernatant of HCT 116 and MCF-7 tumor lysate pulsed DCs whereas the IL-12 concentration showed significant difference (518.2 ± 3.88 and 485.8 ± 6.005 pg/mL in HCT 116 and MCF-7, respectively, *P* < 0.001) at ratio 1:3 (DC: APO) in comparison with the immature DCs. Furthermore, the results showed that the release of IL-6 was increased by HCT 116 (1167 ± 2.59 pg/mL) and MCF-TLY pulsed DC (1493 ± 1.24 pg/mL) at ratio 1:3 more than at 1:1 which was significantly different in comparison with the non-pulsed DCs and also from LPS-only stimulated DCs (1050 ± 3.19 pg/mL). In contrast, the most profound effect was observed in the suppression of IL-10 release in 1:1 as well as 1:3 loaded DCs since the cells were matured and shifted towards the production of IL-12. Interestingly, the incorporation and uptake of tumor lysate influenced the cytokines release profile of DCs which then were stimulated with LPS. In particular, there was a statistically significant increase in IL-12 P40 as well as IL-6 production and a remarkable reduction in IL-10 release (Fig. 5).

### Increase in the migration potential of tumor lysate pulsed DCs

**Fig. 6:**
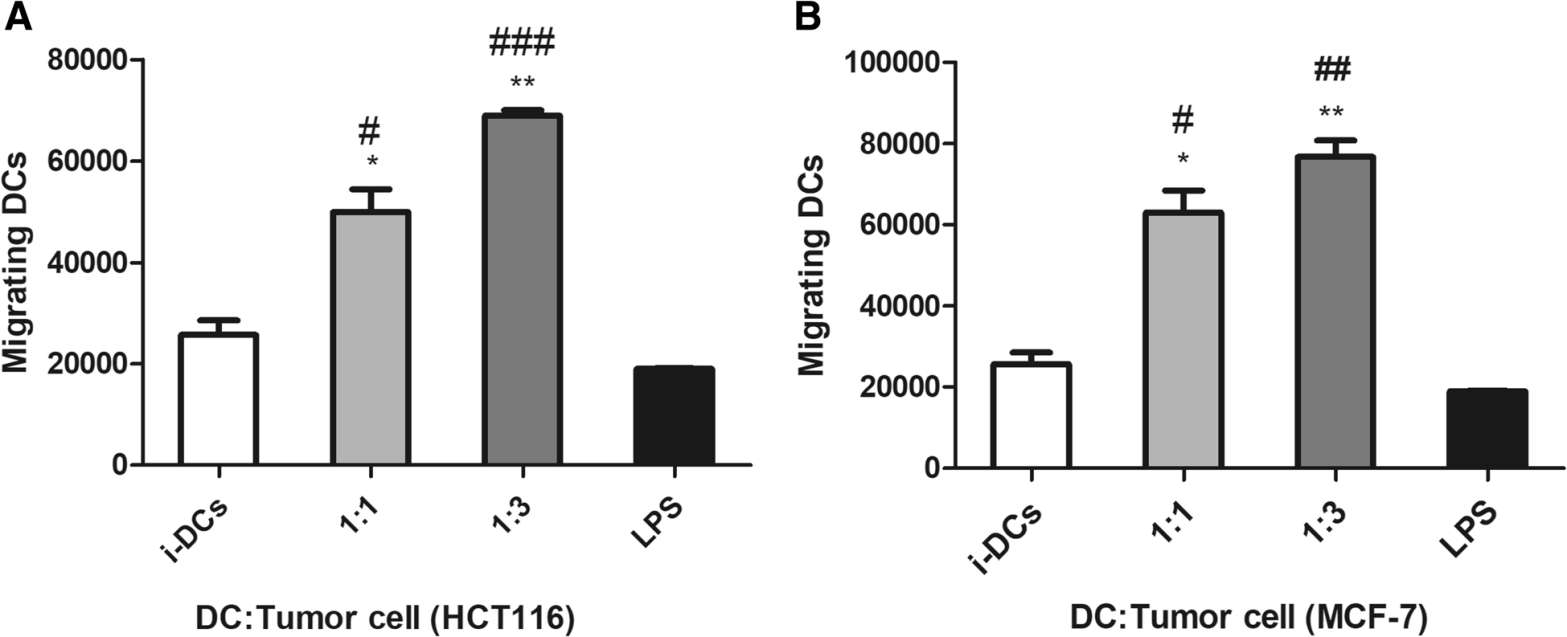
Enhancement in the migration capacity of tumor lysate pulsed dendritic cells. * P < 0.05, ** *P* < 0.01 and *** *P* < 0.001 for **a** HCT-116-TLY-DCs and **b** MCF-7-TLY treated DCs versus iDCs. # Indicated significant difference (# *P* < 0.05, ## *P* < 0.01 and *### P* < 0.001) of TLY-DCs versus LPS-stimulated DCs. Data were statistically analyzed by one-way ANOVA followed by post Dunnett’s test. Each point represents the mean ± SEM of three experiments comprising three different healthy donors. iDCs, immature dendritic cells; TLY, tumor lysate; LPS, Lipopolysaccharide

Migration of matured DCs to lymph node is an essential step in the development of DCs–based vaccine. Thus, the migration potential of TLY-loaded DCs was evaluated at 1:1 and 1:3 ratios (DC: APO). The results demonstrated that both HCT 116 and MCF-7 TLY pulsed DCs displayed an enhanced migration capacity toward LN chemo-attractant CCL21 (250 ng/mL) up to 3–4 times in comparison with unloaded as well as LPS-stimulated DCs only. This finding indicated the DCs maturation. Stimulation of DCs with LPS alone did not improve the migration capacity of the cells; therefore, loading of DCs with tumor lysate dramatically induced the maturation and migration of the DCs towards the lymph node (Fig. 6).

### Allogeneic T cell stimulation capacity

**Fig. 7:**
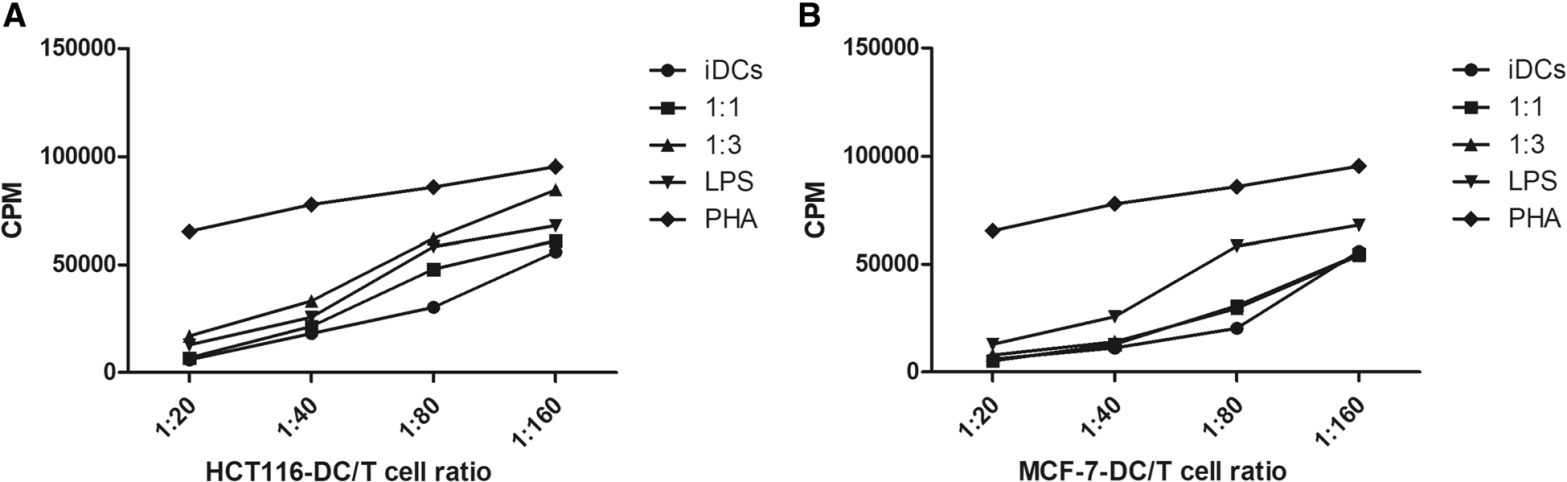
Increase of the allogeneic T cell proliferation capacity of HCT 116 tumor lysate pulsed dendritic cells. iDCs were pulsed with **a** HCT116 TLY and **b** MCF-7 TLY at 1:1 and 1:3 ratios. PHA was used as a positive control. The data are given in mean ± SEM of three experiments comprising three different healthy donors. iDCs, immature dendritic cells; TLY, tumor lysate; LPS, Lipopolysaccharide; CPM, count per minute; PHA, Phytohemagglutinin

T cell proliferation assay was performed to determine the capacity of *P. amarus*-generated tumor lysate pulsed DCs to stimulate allogeneic T cell proliferation. This assay was carried out to identify the stimulation capacity of tumor lysate loaded DCs (1:1 and 1:3) to T cell response as a marker of DCs stimulation and maturation. The lymphocyte proliferation was studied using liquid scintillation counter. The results demonstrated that T cell proliferation capacity of HCT-116 mature DCs was significantly increased in comparison with unloaded immature DCs as well as LPS-stimulated DCs. However, MCF-7 TLY showed inhibitory impact on T cell proliferation capacity of the loaded DCs. This finding indicated that in vitro loading of immature DCs with PA–HCT-116 TLY improved the T cell stimulation capacity that was attributed to the maturation and up-regulation in the surface expression of co-stimulatory molecules CD 86 and CD 83 mainly in HCT116 TLY-DCs (Fig. 7).

### Effect of the tumor lysate on the gene expression profile of dendritic cells

**Fig. 8:**
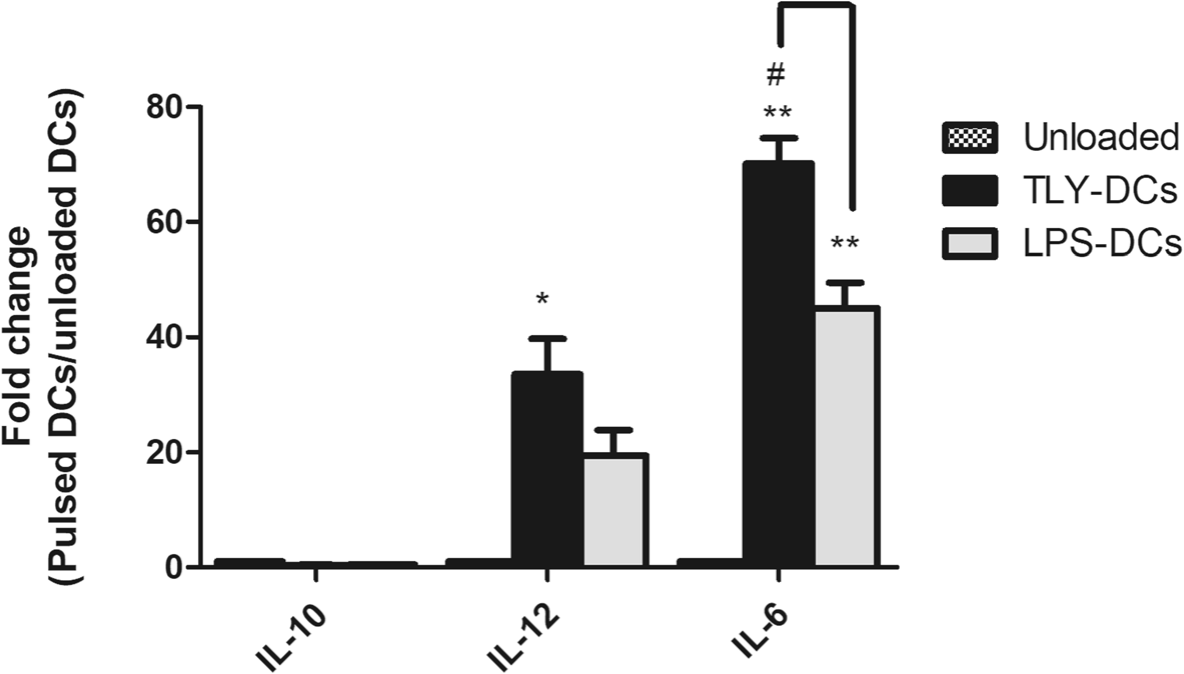
Alteration in the gene expression profile of IL-10, IL-12 P40 and IL-6 before and after tumor lysate pulsing of dendritic cells. The data show the fold change between loaded /unloaded DCs. The data were normalized to GAPDH mRNA expression level. #*P* < 0.05 represents the significant difference from LPS -DCs. *P < 0.05, **P < 0.01, and ***P < 0.001 represent significance to unloaded DCs. The data are given in mean value ± SEM of three experiments comprising three different healthy donors. iDCs, immature dendritic cells; TLY, tumor lysate; LPS, Lipopolysaccharide

Based on the above results, the ability of HCT116-PA-generated TLY to modulate the gene expression profile of the pulsed dendritic cells was elucidated. The mRNA expression of IL-12 P40, IL-6, and IL-10 were studied by RT-qPCR relative to GAPDH mRNA expression level. The result demonstrated the significant up-regulation in the gene expression level of both IL-12 P40 and IL-6 in HCT 116-TLY pulsed DCs at 1: 3 ratios in comparison with unloaded DCs as well as LPS-stimulated only DCs (Fig. 8). In contrast, the expression of IL-10 gene was undetectable in the non-pulsed as well as TLY pulsed DCs cells. This finding supported that TLY pulsed DCs retained their capacity to produce pro-inflammatory cytokines, which were able to trigger their migration into lymph nodes to induce an effective immune response against the recognized tumor antigens.

## Discussion

Dendritic cells are the most commanding and professional APCs that implement a key role in cancer immunotherapy. Several studies referred that DCs pulsed with apoptotic tumor cells lysate have been used successfully to induce tumor vaccination [27]. Furthermore, the using of whole tumor cell lysate represents a rich source of antigens with epitopes for CD8^+^ cytotoxic T cells (CTLs) as well as CD4^+^ T helper cells that generate strong and a long-lasting antitumor immune response [28, 29]. There are several studies that demonstrated the effectiveness of different types of tumor cell death on the stimulation of DCs. Apoptosis is the programmed cell death that is characterized by the loss of the plasma membrane asymmetry and externalization of phosphatidylserine (PS) to the outer leaflet of the cell membrane [30]. DCs express PS receptors that were found to perform an essential role in the uptake of apoptotic cells [31]. However, irradiated tumor cells that showed approximately 30 to 35% of apoptosis, inhibited DCs maturation through the downregulation of MHC II and CD 86 expression as well as suppression of IL-12, TNF α and IL-6 release [32]. Although the danger theory suggests that the necrotic cell death elicits trigger signals to initiate an effective innate immune response [33], many studies reported the immunosuppressive effect of the necrotic cell material generated by freeze and thaw cycles on the immune system [34]. Therefore, the process of induction of mode of cell death can figure the immunogenicity of the generated tumor lysate. Accordingly, in this study we aimed to use *P. amarus* to induce programmed cell death in HCT 16 human colon cancer and MCF-7 human breast cancer cell lines, create a collection of tumor-associated antigens (TAAs) that could activate DCs and evaluate whether this priming process would induce the antigen presenting and processing as well as the cellular immune function of the primed dendritic cells.

HPLC analysis revealed the presence of lignans and polyphenolic compounds mainly gallic acid, ellagic acid and geraniin that have been reported by previous studies to inhibit the cell proliferation and induce apoptosis in different tumor cell types [35–37]. *P. amarus* successfully induced apoptosis in HCT 116 and MCF-7 at 1000 μg/mL with 80.55 ± 2.05% and 75.9 ± 0.6%, respectively, after 24 h and this was detected with Annexin V FITC dual staining kit.

The immature monocyte-derived DCs were generated ex vivo and pulsed with tumor lysate at ratios 1:1 and 1:3 (DCs: APO) that were found to be non-toxic to the DCs. It was clear from our data that ratio 1:1 showed lower stimulatory effect on the DCs activities. Furthermore, 1:3 ratio has been commonly used in the previous clinical trials^.^ [38, 39]. For complete maturation of DCs, the loaded cells were subsequently stimulated with LPS (1 μg/mL). The efficiency and safety of LPS as a maturation stimulus for DCs were already established in various clinical studies with almost no or limited side effects [40, 41]. The data showed that the loaded DCs with tumor lysates from human colon cancer HCT 116 and breast cancer MCF-7 upregulated the surface expression of maturation markers including, MHC I, MHC II as well as adhesion molecule CD 11c. The expression of co-stimulatory molecules CD 86 and CD 83 in HCT 116–TLY DCs was markedly increased and correlated with their high T-cell proliferation capacity. In contrast, MCF-7 tumor lysate reduced the T cell proliferation capacity of loaded DCs followed the engulfment of tumor lysate. Previous studies reported that soluble factors produced by the tumor cells may participate in the attenuation of the DCs allostimulatory function such as transforming growth factor β and other tumor-derived lipids without affecting the DCs phenotypic expression of MHC II [42]. Therefore, T cell proliferation activity of tumor lysate-challenged DCs is an important determinant in development of an effective DCs-based vaccine that should be monitored before the clinical studies. In order to confirm that the generated DCs ex vivo were fully functioning as an APC, we studied their antigen uptake capacity towards HCT 116 and MCF-7 cancer cells debris stained with CFSE fluorescence dye. DCs were found to display a strong phagocytic activity towards tumor cell remnants up to 85 and 88% for both cell lines. Generally, mature DCs are characterized by reduced capacity of further antigen uptake [43], thus the endocytic activity of loaded DCs for FITC- dextran was evaluated, and it was impaired as a hallmark of DCs activation and maturation. However, immature DCs could strongly incorporate FITC-dextran up to 98.1 ± 1.6%.

The cytokines profile of DCs was studied by evaluation of their release in the supernatant in unloaded, tumor lysate pulsed DCs as well as LPS-stimulated only DCs. Our data showed the significant upregulation in IL-12 and IL-6 release in compare with unloaded and also LPS-stimulated DCs as a characteristic of DCs maturation. On the other hand, the production of the anti-inflammatory cytokine IL-10 in tumor lysate-pulsed DCs was strongly attenuated following the engulfment of tumor lysate. IL-12 is involved in the differentiation of naive T cell into Th1 phenotype [44], in addition to its ability to induce the proliferation and growth of T lymphocytes as well as the release of TNFα and INF γ. Besides, IL-12 enhances the cytotoxic activity of CD 8^+^ T lymphocytes and natural killer cells (NK cells) against the tumor cells. Thus, this finding revealed that *P. amarus*-generated tumor lysate induced the release of IL-12 by pulsed DCs that possesses an important function in the regulation of T lymphocyte-induced antitumor immune response. IL-6 was reported to enhance CD8^+^ T-cell proliferation in vitro [45, 46] and in vivo. Furthermore, IL-6 induced the survival of naïve T cells [47] and participated in a complete and effective in vivo cytotoxic CD8^+^ T-cell response. In contrast, IL-10 is an anti-inflammatory cytokine that induces immunosuppressive response which enables the tumor cells to evade the immune control [48]. Additionally, it is associated with the generation of T _reg_ lymphocytes that inhibit the antitumor immune response and support the tumor growth and propagation [49]. The results for the cytokines release have been confirmed by determination of their mRNA expression levels and the transcriptional change in their encoding genes before and after tumor lysate challenge. In this study, the effect of *P. amarus*-generated tumor lysate on the migration capacity of the DCs was investigated toward CCL21 LN-secreted chemokine. The data detected the obvious enhancement in the migration potential of the loaded DCs with tumor lysate in the presence of maturation signal from LPS, in comparison with unloaded DCs. However, LPS-stimulated only DCs demonstrated lower migration capacity than pulsed DCs since the earlier studies stated that the maturation of DCs using LPS as well as INF γ did not improve the migration ability of the cells and also could not migrate well [50]. Maturation of DCs mainly via antigen uptake and presentation is accompanied by their migration to lymphoid tissue in order to impulse naïve T-lymphocytes. Therefore, this finding suggested that TLY-pulsed DCs were activated and matured.

## Conclusions

In conclusion, the present study demonstrated that *P. amarus*-generated tumor lysate induced the expression of maturation markers mainly MHC I, CD 86, CD 83 as well as the adhesion molecule CD11c in pulsed DCs. Furthermore, it induced IL-12 and Il-6 cytokines release and suppressed IL-10 production. Additionally, DCs migration and T cell proliferation allostimulatory capacities were highly stimulated. Taken together, our data revealed that whole tumor lysate generated by *P. amarus* induced the maturation of DCs and their capacity to stimulate an efficient in vitro antitumor immune response. Thus, we present a novel in-vitro DCs-based vaccine model that has been developed against colon and breast cancer by using a natural immunomodulatory, *P. amarus*-induced apoptotic tumor cells. Nevertheless, further in vivo studies in different animal models, pharmacokinetic and pre-clinical investigations are needed before human studies can be carried out on the generated DCs based vaccine.

## Abbreviation

APC: Antigen presenting cells
ATCC: American type culture collection
CFSE: 5, 6-carboxyfluorescein diacetate succinimidyl ester
CTLs: Cytotoxic T lymphocytes
DCs: dendritic cells
DMEM: Dulbecco’s modified Eagle’s medium
ELISA: enzyme-linked immunosorbent assay
FBS: fetal bovine serum
FITC: Fluorescein isothiocyanate
GM-CSF: Granulocytes-macrophage colony stimulating factor
HPLC: High performance liquid chromatography
IL-4: Interleukin 4
MHC: Major histocompatibility complex
NK: Natural killer cells
PA: *Phyllanthus amarus*
PBMC: peripheral blood monocytes
PHA: Phytohaemagglutinin
PS: Phosphatidylserine
TAA: Tumor associated antigens
TLY: Tumor lysate
TNF-α: Tumor necrosis factor-alpha
